# Supplementary material for: Graph neural network surrogates to leverage mechanistic expert knowledge towards reliable and immediate pandemic response
Source: Sci Rep. 2026 Feb 13;16:6361. doi: 10.1038/s41598-026-39431-5 (PMC12904856; doi:10.1038/s41598-026-39431-5)
Supplement: Supplementary file 1 — Supplementary Information. [file 41598_2026_39431_MOESM1_ESM.pdf]

Supplementary Information

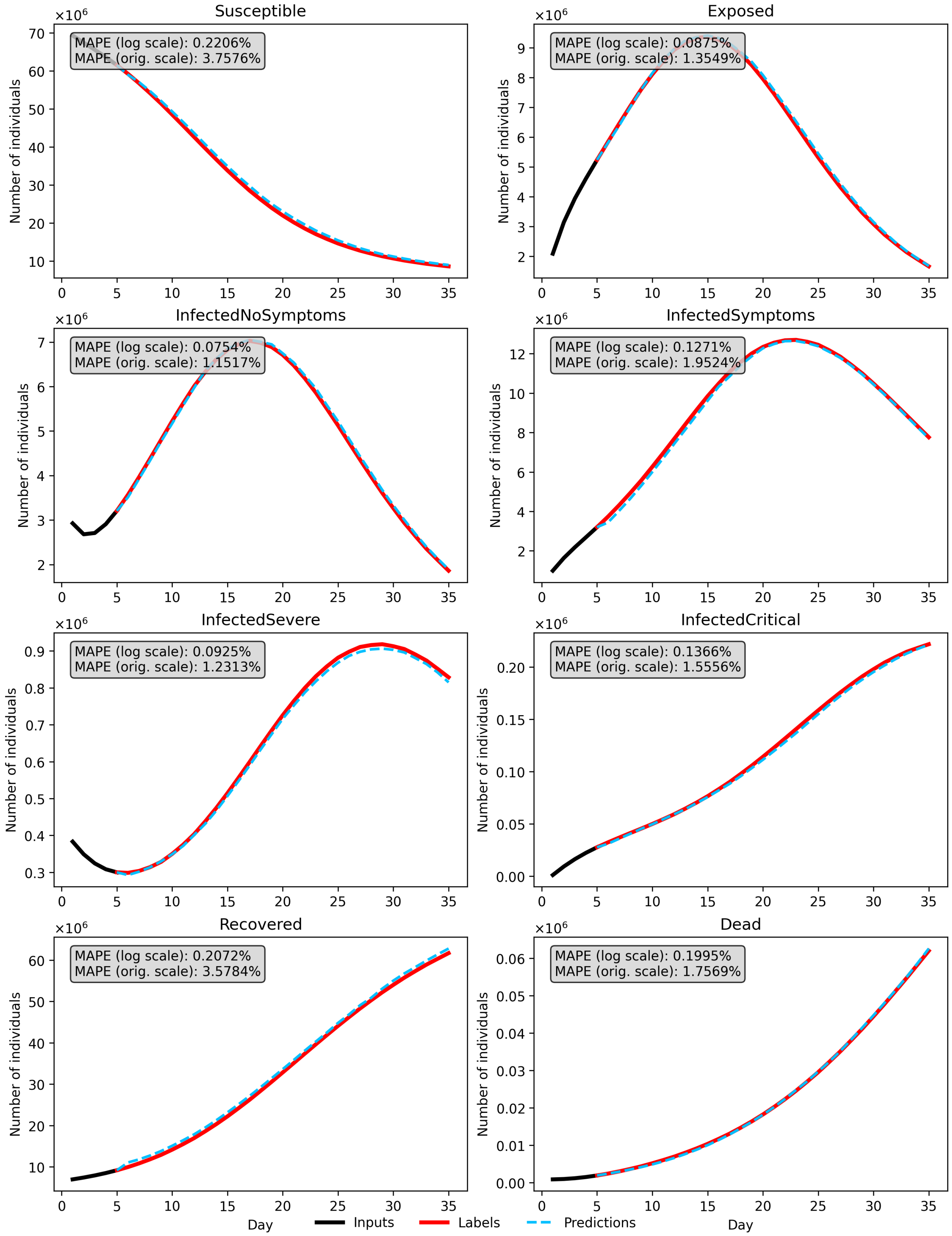

Supplementary Figure 1: LSTM predictions for the first trajectory of the model without stratification into age groups and corresponding MAPE values. The particular prediction was chosen randomly and does neither represent a very good nor very bad prediction, it only allows to visually assess the trajectories against the corresponding MAPE values.

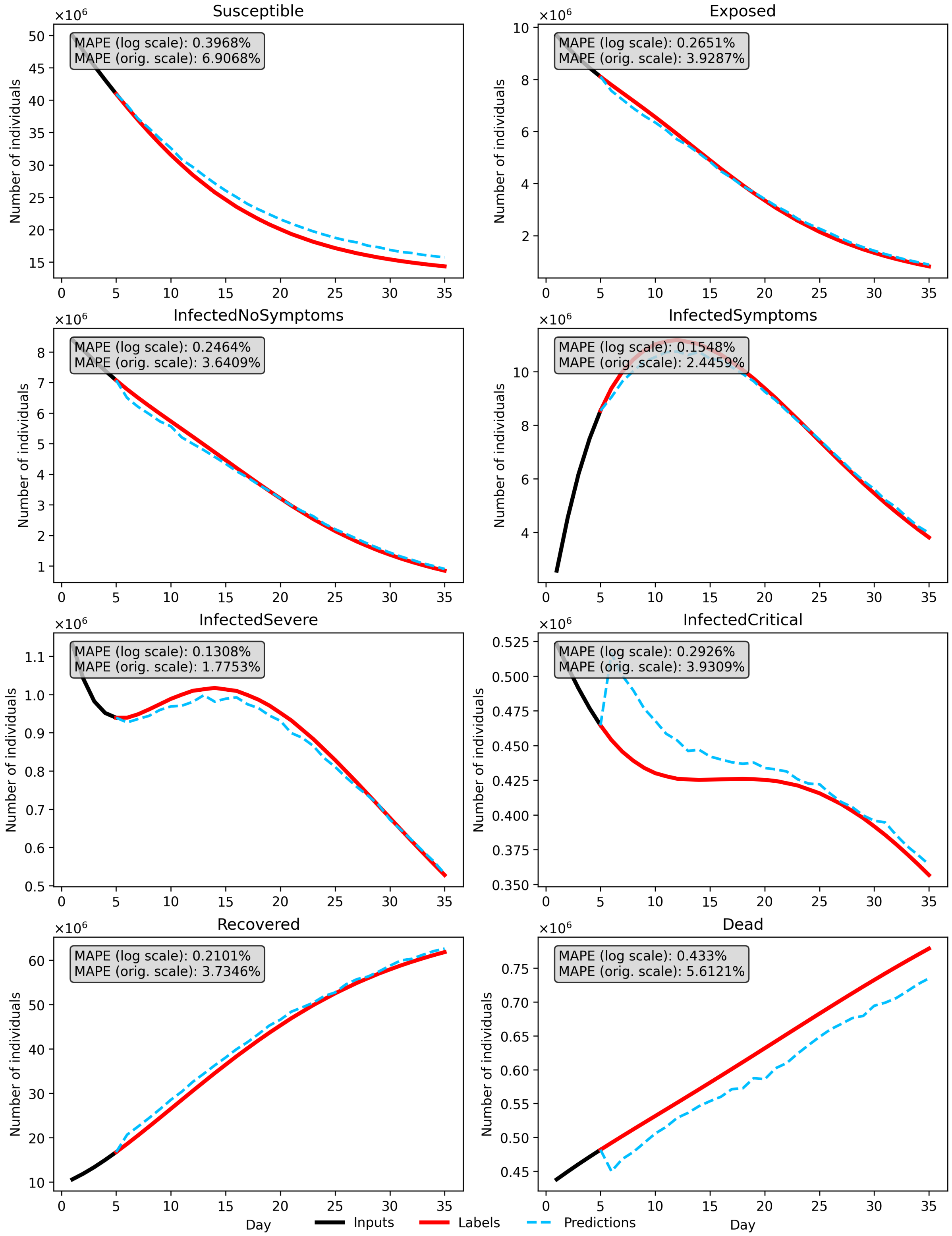

Supplementary Figure 2: LSTM predictions for the first trajectory of the model with stratification into age groups and corresponding MAPE values (summed over all age groups). The particular prediction was chosen randomly and does neither represent a very good nor very bad prediction, it only allows to visually assess the trajectories against the corresponding MAPE values.
